# m^6^A binding protein YTHDF2 in cancer

**DOI:** 10.1186/s40164-022-00269-y

**Published:** 2022-04-05

**Authors:** Xiaomin Chen, Xiangxiang Zhou, Xin Wang

**Affiliations:** 1 Department of Hematology, Shandong Provincial Hospital, Cheeloo College of Medicine, Shandong University, No.324, Jingwu Road, Jinan, 250021 Shandong China; 2 Department of Hematology, Shandong Provincial Hospital Affiliated to Shandong First Medical University, Jinan, 250021 Shandong China; 3 School of Medicine, Shandong University, Jinan, 250012 Shandong China; 4 Shandong Provincial Engineering Research Center of Lymphoma, Jinan, 250021 Shandong China; 5 Branch of National Clinical Research Center for Hematologic Diseases, Jinan, 250021 Shandong China; 6 National Clinical Research Center for Hematologic Diseases, The First Affiliated Hospital of Soochow University, Suzhou, 251006 China

**Keywords:** YTHDF2, m^6^A, Cancer, Mechanism, Prognosis

## Abstract

YT521-B homology domain family member 2 (YTHDF2) is an N^6^-methyladenosine (m^6^A)-binding protein that was originally found to regulate the stability of mRNA. Growing evidence has shown that YTHDF2 can participate in multifarious bioprocesses, including embryonic development, immune response, and tumor progression. Furthermore, YTHDF2 is closely associated with the proliferation, apoptosis, invasion, and migration of tumor cells, suggesting its significant role in cancers. YTHDF2 primarily relies on m^6^A modification to modulate signaling pathways in cancer cells. However, the expression and function of YTHDF2 in human malignancies remain controversial. Meanwhile, the underlying molecular mechanisms of YTHDF2 have not been elucidated. In this review, we principally summarized the biological functions and molecular mechanisms of YTHDF2 in tumors and discussed its prognostic and therapeutic values.

## Introduction

RNA epitranscriptomics has been found to play key roles in numerous cellular functions and has attracted increasing attention. Presently, there have been more than 100 types of chemical modifications of RNA found in various cells [1]. N^6^-methyladenosine (m^6^A) is considered to be the most prevalent and ample internal transcription modification in eukaryotic messenger RNAs (mRNAs), microRNAs (miRNAs), and long noncoding RNAs (lncRNAs) [2, 3].

The methylation modification of m^6^A has been confirmed to be dynamic and reversible, involving methyltransferase “writers”, demethylase “erasers” and methylated reading protein “readers” [4]. For example, methyltransferase-like 3 (METTL3) and METTL14 can form a steady heterodimer complex. They shape the m^6^A methyltransferase complex (MTC) along with their accessory factors Wilms tumor 1-associated protein (WTAP), Vir like m^6^A methyltransferase associated (VIRMA/KIAA1429), RNA binding motif protein (RBM) 15/15b, zinc finger CCCH-Type containing 13 (ZC3H13), and HAKAI [5–10]. These factors act as m^6^A "writers" and collectively catalyze m^6^A modification. “Erasers”, including fat mass and obesity-associated protein (FTO) and alkB homolog 5 (ALKBH5), could dislodge the methyl code of m^6^A modification from target RNA [11, 12]. “Readers” include YT521-B homology (YTH) domain-containing protein, eukaryotic initiation factor 3 (eIF3), insulin-like growth factor 2 mRNA binding protein families (IGF2BPs), and heterogeneous nuclear ribonucleoprotein protein families (HNRNPs). They can recognize and bind to the site of the m^6^A modification and engender functional signals [13–17].

**Fig. 1:**
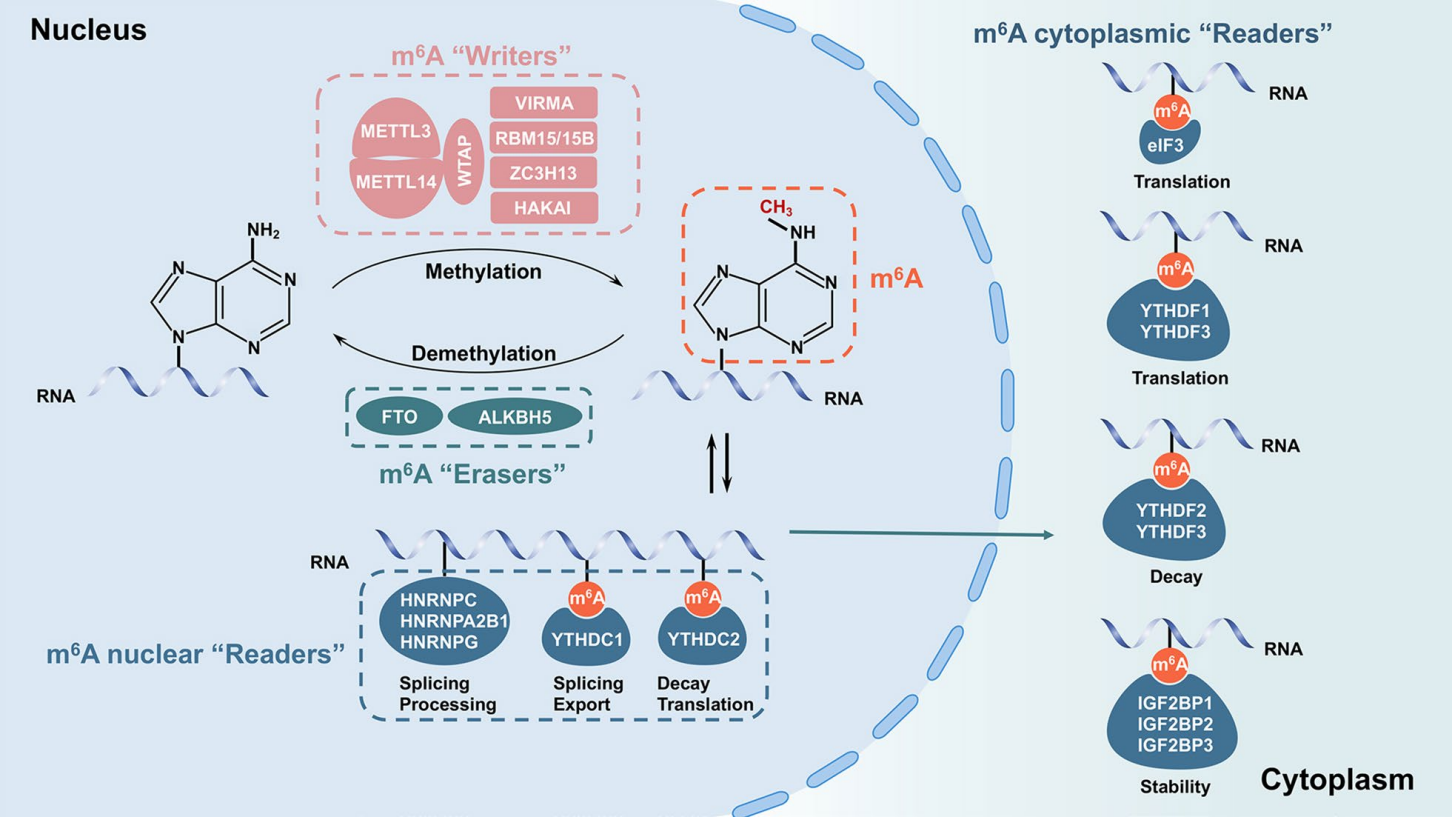
The schematic of m^6^A modification mechanism. METTL3 and METTL14 shape the m^6^A methyltransferase complex along with WTAP, VIRMA, KIAA1429, RBM15/15b, ZC3H13, and HAKAI, serving as m^6^A "writers". FTO and ALKBH5 shape the m^6^A demethylase, serving as m^6^A "erasers". YTHDF1/2/3, eIF3, IGF2BP1/2/3, and HNRNPs shape the m^6^A modification binding protein, serving as m^6^A “readers”

The YTH domain protein family, including YTHDF1, YTHDF2, YTHDF3, YTHDC1, and YTHDC2, has been validated as a direct m^6^A “reader” [18, 19]. Furthermore, YTHDF1, YTHDF2, and YTHDF3 primarily recognize and bind to the site of m^6^A modification in the cytoplasm, while YTHDC1 and YTHDC2 act in the nucleus [20, 21]. According to previous reports, YTHDF2, YTHDF3, and YTHDC2 functioned to accelerate the degradation of target mRNAs, YTHDF1, YTHDF3, and YTHDC2 increased the translation of target mRNAs, and YTHDC1 regulated the splicing and nuclear export of target mRNAs [22, 23]. The eIF3 protein is capable of binding to the site of m^6^A modification on the 5’-UTR of mRNA, thereby accelerating the translation of RNA [24]. The details of the currently known m^6^A modification mechanism are shown in Fig. 1.

Methylated m^6^A actively participates in many vital physiological processes, such as stem cell differentiation and pluripotency, embryonic development, circadian rhythm, and DNA damage response. With consecutive studies on the function and mechanism of m^6^A, it has been shown that the progression of several types of cancer can be affected by the abnormal expression of m^6^A methylation-related proteins [25–28]. Furthermore, methylated m^6^A is involved in the biological processes of cancer cells, including cell self-renewal and differentiation, the pluripotency of cancer stem cells (CSCs), cell proliferation, metastasis, and tumor immunity [29–31].

It is known that m^6^A modification can recruit particular “reader” proteins or alter the structure of mRNA to modulate the processing, stability, and translation of mRNA [32, 33]. Among them, YTHDF2, the binding protein of m^6^A, was the first discovered and most efficient m^6^A “reader” [29]. It was reported that YTHDF2 could regulate mRNA degradation and cell viability [16, 34]. The interaction binding site between YTHDF2 and m^6^A was usually located in the 3’-UTR of mRNA[29]. However, emerging evidence suggested that YTHDF2 specifically bound to mRNA bearing m^6^A methylation markers at the 5’-UTR, which subsequently facilitated protein translation [35]. YTHDF2 was reported to present dual functions in tumors by regulating the proliferation and migration of tumor cells [36, 37]. For example, YTHDF2 was upregulated and acted as an oncogene in multiple cancers, including acute myelocytic leukemia (AML), lung cancer, and gastric cancer [38–40]. In contrast, YTHDF2 was also found to be downregulated and served as a tumor suppressor in osteosarcoma and melanoma [31, 41].

Based on the controversial role of YTHDF2 in various cancers, we summarized its expression patterns and molecular mechanisms in tumorigenesis and discussed the potential prognostic and therapeutic value of YTHDF2 in malignant tumors.

## The structure of YTHDF2

**Fig. 2:**
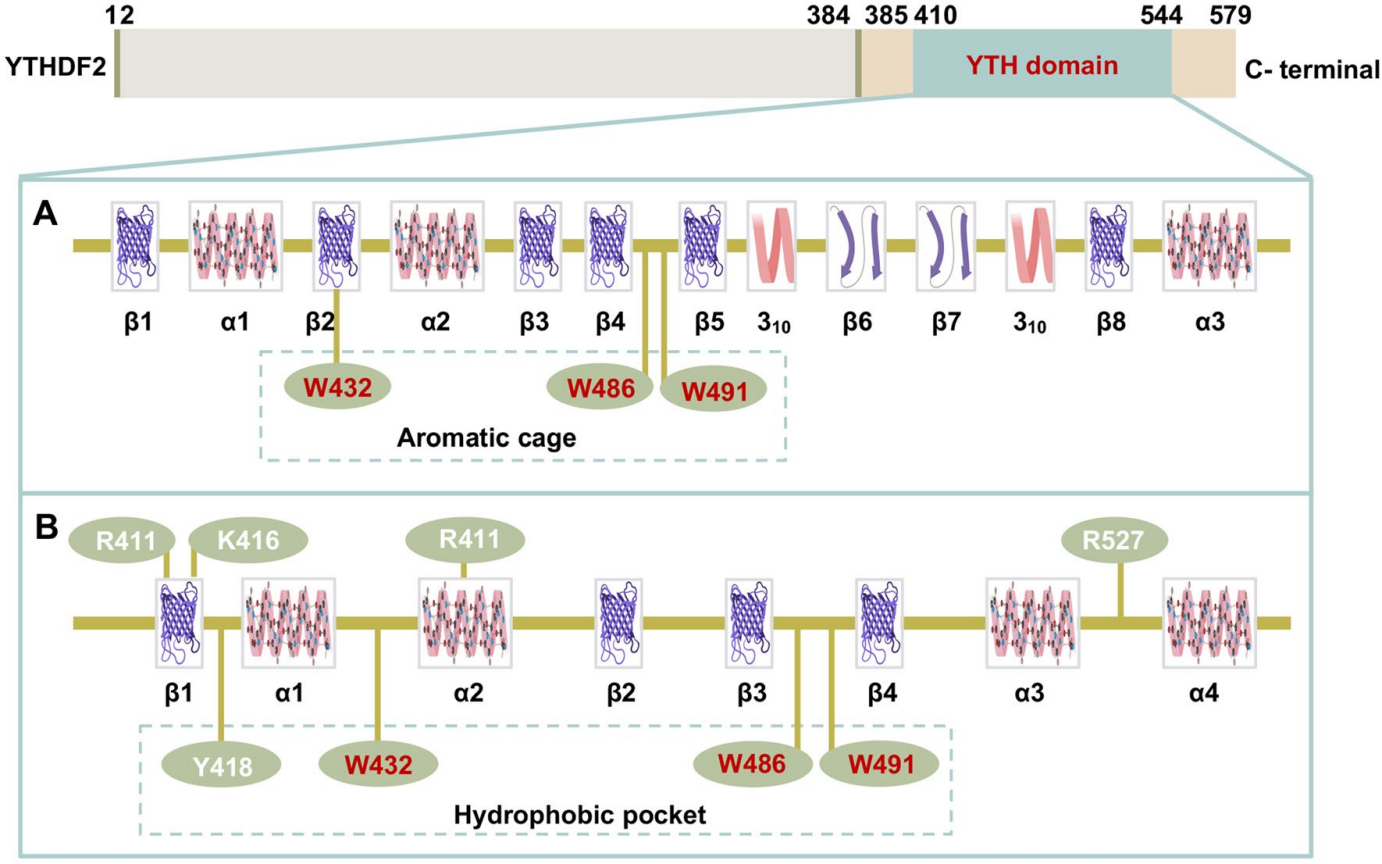
Two hypotheses of YTHDF2 structure. **A** Eight β-strands (β1-β8), three α-helices (α1-α3) and two 3_10_-helices in YTHDF2 domain, with W486, W432, and W491 form an aromatic cage containing m^6^A mononucleotides. **B** The structure of the YTH domain contains four β-sheets (β1–β4), four α-helices (α1–α4), and flanking regions on both sides. The aromatic residues Y418, W432, W486, and W491 form a hydrophobic pocket

YTHDF2 occupies the full length of 579 amino acids (aa), has the regions localized to mRNA processing bodies (aa 2–384), and interacts with m^6^A-containing mRNAs (aa 385–579) that contains the YTH domain (aa 410–544), which includes the m^6^A binding site [16]. A previous study has indicated that the YTH domain of YTHDF2 is globularly folded with a central core consisting of eight β-strands (β1-β8), three α-helices (α1-α3), and two 3_10_-helices. Furthermore, residues W486 in the β4-β5 loop, W432 in the β2 strand, and W491 in the β4-β5 loop form an aromatic cage containing m^6^A [42] (Fig. 2A). Interestingly, it was also proved that the YTH domain of YTHDF2 is a globular fold with a four-stranded β-sheet (β1-β4), four α-helices (α1-α4), and flanking regions on both sides. A hydrophobic pocket is formed by the aromatic residues Y418, W432, W486, and W491 [43, 44] (Fig. 2B).

## Expression pattern and function of YTHDF2 in human cancers

The expression pattern of YTHDF2 has been confirmed in numerous studies, and the expression level of YTHDF2 has been found to vary in different types of cancer. In most cases, the expression of YTHDF2 is upregulated in tumor tissues in comparison with normal tissues, and YTHDF2 plays an oncogenic role in these types of cancers. Nevertheless, even within the same cancer type, several studies have yielded opposite results. The detailed expression levels of YTHDF2 in various cancers are shown in Table 1. The targets of YTHDF2 and their functions in cancers are shown in Table 2.

**Table 1:** The role of YTHDF2 in cancers

Cancer type	Expression	Role	Function in cancer	Molecular mechanism	Year
GC	Upregulated	Oncogene	Facilitating proliferation, invasion and migration	Mediating the degradation of PTEN mRNA to activate PI3K/AKT signaling pathway	2019
	Downregulated	Tumor suppressor	Inhibiting proliferation, migration and prolonging OS	Regulating FOXC2 Signaling	2019, 2020
CRC	Upregulated	Oncogene	Facilitating proliferation	Regulated by miR-145 and regulating Wnt/β-catenin pathway	2021
	Downregulated	Tumor suppressor	Restraining proliferation and metastasis	Modulating the degradation of XIST	2020
	–	Tumor suppressor	Prolonging OS	–	2020
Liver cancer	Upregulated	Oncogene	Enhancing proliferation	Regulated by miR-145	2017
			–	Mediating the degradation of SOCS2 mRNA	2018
			Shortening OS	–	2020
			Promoting liver cancer stem cell phenotype and metastasis	Mediating the translation of OCT4	2020
	Downregulated	Tumor suppressor	Repressing proliferation and growth	Regulating MAPK/ERK signaling	2018
			Suppressing proliferation, metastasis, tumor inflammation and vascular abnormalities Prolonging OS and RFS	Mediating the degradation of IL11 and serpin E2 mRNAs	2019
PC	Upregulated	Oncogene and tumor suppressor	Enhancing proliferation and inhibiting invasion, adhesion, migration and EMT	Regulating Hippo signaling	2017
	–	Oncogene	–	Mediating the degradation of PER1 mRNA	2020
	–	Tumor suppressor	Inhibiting proliferation and migration	Modulating the degradation of PIK3CB mRNA to inhibit activation of PI3K/AKT signaling pathway	2020
Lung cancer	Upregulated	Oncogene	Accelerating proliferation and metabolism defect	Mediating the translation of 6PGD to regulate pentose phosphate pathway	2019
		–	Prolonging OS and RFS	–	2020
		Oncogene	Facilitating proliferation, reducing apoptosis, but prolonging OS and RFS	–	2021
		Oncogene	Enhancing cell growth, colony formation and migration	Regulating Wnt/β-catenin pathway	2021
	Downregulated	Tumor suppressor	Suppressing proliferation, invasion, migration and EMT and prolonging OS	Regulating Hippo signaling	2020
Leukemia	Upregulated	Oncogene	Facilitating proliferation, restraining apoptosis and marrow reconstitution	Regulating TNF signaling	2019
			Enhancing proliferation, suppressing apoptosis	Regulating TNF signaling	2021
PTCL-NOS	–	Oncogene	Shortening OS	–	2019
PCa	Upregulated	Oncogene	Promoting proliferation, migration and colony formation, suppressing apoptosis	Mediating the degradation of LHPP and NKX3–1 mRNAs	2020
			Enhancing proliferation, migration and invasion, reducing apoptosis	Regulated by miR-495 and mediating the degradation of MOB3B mRNA	2020
Bladder cancer	Upregulated	Oncogene	Facilitating migration	Mediating the degradation of SETD7 and KLF4 mRNAs	2020
ccRCC	Downregulated	Tumor suppressor	Prolonging OS	–	2020
CC	–	Oncogene	Promoting proliferation, migration and invasion	Modulating the degradation of GAS5 mRNA	2019
Ovarian cancer	Upregulated	Oncogene	Promoting proliferation and colony formation, inhibiting apoptosis	Mediating the degradation of BMF mRNA	2021
Breast cancer	Upregulated	Oncogene	Facilitating proliferation, inhibiting apoptosis and cell cycle arrest	Modulating the degradation of PRSS23 mRNA	2021
Osteosarcoma	–	Tumor suppressor	Inhibiting proliferation and metastasis	Regulating the degradation of PVT1 mRNA	2020
Melanoma	Upregulated	Oncogene	Promoting proliferation, migration and colony formation	Mediating the degradation of PER1 and TP53 mRNAs	2021
	Downregulated	Tumor suppressor	Suppressing proliferation and migration	Regulating the degradation of PD-1, CXCR4, and SOX10 mRNAs	2019
GBM	Upregulated	Oncogene	Maintaining glioblastoma stem cells	Modulating the stability of MYC and VEGFA mRNAs	2020
			Facilitating proliferation, invasion, and tumorigenesis	Mediating the degradation of LXRA and HIVEP2 mRNAs	2021
			Promoting proliferation and migration	Regulating the degradation of UBXN1 mRNA	2021
HNSCC	Upregulated	Oncogene	Shortening OS	–	2020

“-” not illustrated, *GC *Gastric cancer, *CRC* Colorectal cancer, *PC* Pancreatic cancer, *PTCL-NOS* Peripheral T-cell lymphoma, not otherwise specified, *PCa* Prostate cancer, *ccRCC* Clear cell renal cell carcinoma, *CC* Cervical cancer, *GBM* Glioblastoma, *HNSCC* Head and neck squamous cell carcinoma

**Table 2:** The targets of YTHDF2 and their functions in cancers

Cancer type	Target	Gene description	Role	References
GC	PTEN	Phosphate and tension homology deleted on chromosome ten	Tumor suppressor	[39]
	FOXC2	Forkhead box protein C2	Oncogene	[46]
CRC	miR-1625	microRNA 6125	Tumor suppressor	[49]
	GSK3β	Glycogen synthase kinase 3 beta	Tumor suppressor	[49]
	XIST	X inactivate-specific transcript	Oncogene	[47]
Liver cancer	miR-145	microRNA 145	Tumor suppressor	[55]
	SOCS2	Suppressor of cytokine signaling 2	Tumor suppressor	[54]
	OCT4 (POU5F1)	POU class 5 homeobox 1	Oncogene	[53]
	EGFR	Epidermal growth factor receptor	Oncogene	[36]
	Serpin E2	Serpin peptidase inhibitor clade E member 2	Oncogene	[50]
	IL11	Interleukin-11	Oncogene	[50]
PC	YAP	YES-associated protein	Oncogene	[56]
	PER1	Period circadian regulator 1	Tumor suppressor	[58]
	PIK3CB	Phosphoinositide-3-kinase catalytic beta	Oncogene	[57]
Lung cancer	6PGD	6-phosphogluconate dehydrogenase	Oncogene	[40]
	AXIN1	axin 1	Tumor suppressor	[62]
Leukemia	TNFRSF1B	TNF receptor superfamily member 1b	Tumor suppressor	[38]
PCa	LHPP	Phospholysine phosphohistidine inorganic pyrophosphate phosphatase	Tumor suppressor	[66]
	NKX3–1	NK3 homeobox 1	Tumor suppressor	[66]
	miR-495	microRNA 495	Tumor suppressor	[67]
	MOB3B	MOB kinase activator 3B	Tumor suppressor	[67]
Bladder cancer	SETD7	SET domain containing 7	Tumor suppressor	[65]
	KLF4	Kruppel like factor 4	Tumor suppressor	[65]
CC	GAS5	Growth arrest specific 5	Tumor suppressor	[73]
Ovarian cancer	BMF	Bcl2 modifying factor	Tumor suppressor	[72]
Breast cancer	PRSS23	Serine protease 23	Tumor suppressor	[71]
Osteosarcoma	PVT1	Plasmacytoma variant translocation 1	Oncogene	[41]
Melanoma	TP53	Tumor protein P53	Tumor suppressor	[75]
	PD-1	Programmed death 1	Oncogene	[31]
	CXCR4	C-X-C motif chemokine receptor 4	Oncogene	[31]
	SOX10	SRY-Box Transcription Factor 10	Oncogene	[31]
GBM	MYC	MYC proto-oncogene	Oncogene	[76]
	VEGFA	Vascular endothelial growth factor A	Oncogene	[76]
	LXRA	Liver X receptors A	Tumor suppressor	[77]
	HIVEP2	HIVEP Zinc Finger 2	Tumor suppressor	[77]
	UBXN1	UBX domain protein 1	Tumor suppressor	[78]

*GC* Gastric cancer, *CRC* Colorectal cancer, *PC *Pancreatic cancer, *PCa  *Prostate cancer, *CC *Cervical cancer, *GBM *Glioblastoma

### YTHDF2 in digestive system tumors

#### Gastrointestinal cancer

##### Gastric cancer (GC)

Yan et al*.* and Zhang et al*.* elucidated that YTHDF2 was upregulated in GC [39]. Conversely, recent studies demonstrated that YTHDF2 was downregulated in GC [45, 46]. Overexpression of YTHDF2 accelerated the degradation of phosphate and tension homology deleted on chromosome ten (PTEN) mRNA, a remarkable tumor suppressor, which considerably increased the proliferation, invasion, and migration of GC cells [39]. However, through Gene Set Enrichment Analysis (GSEA) and external experiments such as quantification of m^6^A methylation and western blot assay, YTHDF2 was found to be a potential tumor inhibitory factor, and high YTHDF2 expression was correlated with the prolonged survival time of GC patients [45]. Additionally, knockout of YTHDF2 significantly increased the expression of Forkhead box protein C2 (FOXC2), thereby suppressing the proliferation, invasion, and migration of GC cells [46].

##### Colorectal cancer (CRC)

It has been demonstrated that YTHDF2 is downregulated in CRC [47]. Moreover, Yang et al. detected that YTHDF2 inhibited the expression of X inactivate-specific transcript (XIST) in CRC cells, which could accelerate tumor growth and metastasis [47]. Similarly, Zhuang et al. demonstrated that YTHDF2 acted as a protective gene, which led to better overall survival (OS) in rectal cancer patients [48]. In summary, YTHDF2 may play an essential role in the prognosis of CRC. However, the protein levels of YTHDF2 were recently reported to be elevated in CRC tissues in comparison with adjacent normal tissues [49]. Overexpression of YTHDF2 facilitated the proliferation of CRC cells, suggesting that YTHDF2 may play a carcinogenic effect in CRC [49].

##### Hepatocellular carcinoma (HCC)

Hou et al. and Zhong et al. found decreased expression of YTHDF2 in both HCC tissues and HCC cells [36, 50]. Patients with low YTHDF2 expression presented higher TNM, advanced BCLC stage classification, lower OS and relapse-free survival (RFS) rates. Silencing of YTHDF2 accelerated tumor inflammation and vascular abnormalities, thereby promoting the tumor growth, metastasis, and vascular remodeling of liver cancer [50]. The positive expression of YTHDF2 could restrain cell proliferation and tumor growth in mouse xenografts [36]. Therefore, YTHDF2 may play a critical role in inhibiting tumorigenesis and prolonging survival time.

However, by analyzing data from the Cancer Genome Atlas (TCGA) and Gene Expression Omnibus (GEO) databases, Qu et al. demonstrated that most m^6^A-related genes, including YTHDF2, were drastically highly expressed in HCC tissues and hepatoblastoma cells compared with adjacent normal tissues [51–53]. Similarly, Chen et al. found that YTHDF2 was upregulated in liver cancer [54, 55]. Moreover, the overexpression of YTHDF2 resulted in shortened survival time and poor prognosis [51, 52]. Highly expressed YTHDF2 can promote the proliferation and migration of liver cancer cells [54, 55], increase the number of liver cancer stem cells (CSCs), and enhance the tumor burden and lung metastasis in vivo [53]. To conclude, the contrary results may be associated with the heterogeneity of cell lines and tumor tissues. Consequently, further studies are required to obtain an in-depth understanding of the factors affecting gene function in various cell backgrounds.

##### Pancreatic cancer (PC)

Chen et al. illustrated that the expression of YTHDF2 in PC tissues was upregulated, and found that YTHDF2 gradually increased with the elevation of clinical stage. Knockdown of YTHDF2 induced phase arrest of G1 and suppressed the proliferation of PC cells [56]. Nonetheless, YTHDF2 was observed to decrease the invasion, adhesion, migration, and EMT of pancreatic cancer cells [56]. Moreover, bioinformatics analysis and RNA immunoprecipitation (RIP) analysis revealed that YTHDF2 could bind to its target genes and then promote their degradation, resulting in increased or decreased growth of PC cells [57, 58]. Thus, it can be concluded that YTHDF2 acts as both a positive and negative factor in PC, and further investigations are warranted to improve our knowledge of the involved molecular mechanism.

#### YTHDF2 in respiratory tumors

##### Lung cancer

Jin et al. investigated the role of m^6^A-related genes in non-small-cell lung cancer (NSCLC) and discovered that YTHDF2 was downregulated in tumor tissues [59]. Sheng et al. found that YTHDF2 was highly expressed in lung cancer tissues compared with normal lung tissues [40]. Similarly, it was reported that the expression of YTHDF2 was upregulated in patients with lung adenocarcinoma and NSCLC through bioinformatic analysis [60–62]. Interestingly, the elevated expression of YTHDF2 was positively correlated with the OS and RFS of lung cancer patients, which was attributed to YTHDF2 promoting the enrichment of tumor-infiltrating lymphocytes and inhibiting the expression of PD-L1 [60, 61]. Moreover, the upregulation of YTHDF2 significantly increased the proliferation, migration, colony formation, metabolic defects, and pentose phosphate pathway (PPP) flux of lung cancer cells to promote lung cancer growth [40, 62]. However, YTHDF2 significantly suppressed cell proliferation, invasion, migration, and epithelial-mesenchymal transition (EMT) in NSCLC [59]. In conclusion, the function of YTHDF2 in lung cancer is controversial, and its specific role needs to be further clarified.

#### YTHDF2 in hematological malignancies

##### Acute myelocytic leukemia (AML)

Recent studies have investigated the expression patterns of YTHDF2 in primary AML patients [38, 63]. The results demonstrated that YTHDF2 was remarkably upregulated in all clinical AML subtypes and was essential to the initiation and dissemination of AML in both human and mouse models. YTHDF2 was found to abate the half-life of most m^6^A transcripts, which was conducive to the integrality of leukemic stem cell functions. In addition, knockdown of YTHDF2 in human AML cells markedly suppressed proliferation and promoted TNF-mediated apoptosis, while it did not influence loid differentiation and normal hematopoiesis [38, 63]. It was also proved that targeting YTHDF2 increased the number of hematopoietic stem cells and promoted marrow reconstitution. Meanwhile, knockout of YTHDF2 apparently prolonged the survival time in the AML mouse model compared with the control group [38].

##### Peripheral T-cell lymphoma (PTCL)

Recent studies have discovered repeated exacerbating deletions and mutations of a novel gene, YTHDF2, in peripheral T-cell lymphoma, not otherwise specified (PTCL-NOS), which may imply the functional importance of YTHDF2 in the pathogenesis of this disease[64]. PTCL-NOS were based on unique genetic profiles, including several discrete mature T cell tumor subtypes. These tumors also showed alterations in various low-frequency somatics, including YTHDF2 [64]. Hence, these findings may contribute to offering novel designs in molecular classification and patient stratification of PTCL-NOS.

#### YTHDF2 in urinary tumors

It was reported that YTHDF2 was overexpressed in both bladder cancer and prostate cancer  (PCa) [65–67] and was remarkably downregulated in clear cell renal cell carcinoma (ccRCC) [68]. A lack of YTHDF2 could significantly decrease the migration rate and reduce the expression level of related proteins in bladder cancer cells, indicating that YTHDF2 acts as an oncogene in bladder cancer [65]. Moreover, the positive expression of YTHDF2 in prostate cancer patients manifested a high tumor grade [66]. With the knockdown of YTHDF2, the level of m^6^A in PCa cells was drastically increased. Concurrently, it remarkably suppressed cell proliferation, migration, and colony formation ability, and increased cell apoptosis [66, 67]. Therefore, YTHDF2 was found to act as a tumor-promoting factor in PCa. In ccRCC, YTHDF2 was uncovered to be a protective gene by univariate Cox regression analysis [68].

#### YTHDF2 in gynecological reproductive system tumors

Woo et al. and Niu et al. found no significant biological functions of YTHDF2 on ovarian cancer or breast cancer [69, 70]. However, recent studies showed that YTHDF2 was upregulated in ovarian cancer or triple-negative breast cancer (TNBC) [71]. Either overexpression or knockdown of YTHDF2 did not alter the expression level of tumor suppressor genes in breast cancer [69]. Woo et al. found that YTHDF2 was not the reader in the oncogenes of ovarian cancer and breast cancer and did not exert biological function [70]. However, expression of YTHDF2 was recently reported to be elevated in MYC-driven TNBC compared with hormone receptor-positive and human epidermal growth factor receptor 2 positive breast cancers [71]. The deficiency of YTHDF2 significantly reduced proliferation rates of TNBC cell lines, yet increased apoptosis, and G1 checkpoint arrest [71]. The study also indicated that YTHDF2 was crucial to the survival of TNBC cells, while it is dispensable for cells that were less dependent on high expression levels of MYC, suggesting the elusive role of YTHDF2 in breast cancer [71]. Moreover, YTHDF2 deficiency suppressed the proliferation, anchorage-independent growth, and colony-forming ability of ovarian cancer cell lines[72]. The diverse functions of YTHDF2 may depend on the different contexts of cancers or by modulating different target genes. In cervical cancer (CC), knockdown of YTHDF2 significantly increased the expression and stability of GAS5, a tumor suppressor gene, thereby inhibiting the proliferation, migration and invasion of CC cells in vitro and suppressing the tumor growth and metastasis of CC in vivo [73]. The above results revealed the critical role of YTHDF2-mediated epigenetic alterations in CC progression.

#### YTHDF2 in other cancers

YTHDF2 was also found to be upregulated in head and neck squamous cell carcinoma (HNSCC) [74], ocular melanoma [75], and glioblastoma (GBM) [76–78]. It was reported that YTHDF2 participated in regulating cell proliferation, migration, and invasion in vitro and in vivo, which indicated YTHDF2 as a carcinogenic gene in these tumors [75–78]. Other studies have shown that YTHDF2 is expressed at low levels in melanoma and osteosarcoma [31, 41], where YTHDF2 can directly combine and accelerate the degradation of other oncogenes. Low expression of YTHDF2 in these cancers was found to be linked to poor OS in patients, as well as increased tumor size, TNM stage, lymph node, and distant metastasis [31, 41].

## Molecular mechanisms of YTHDF2 in tumorigenesis

**Fig. 3:**
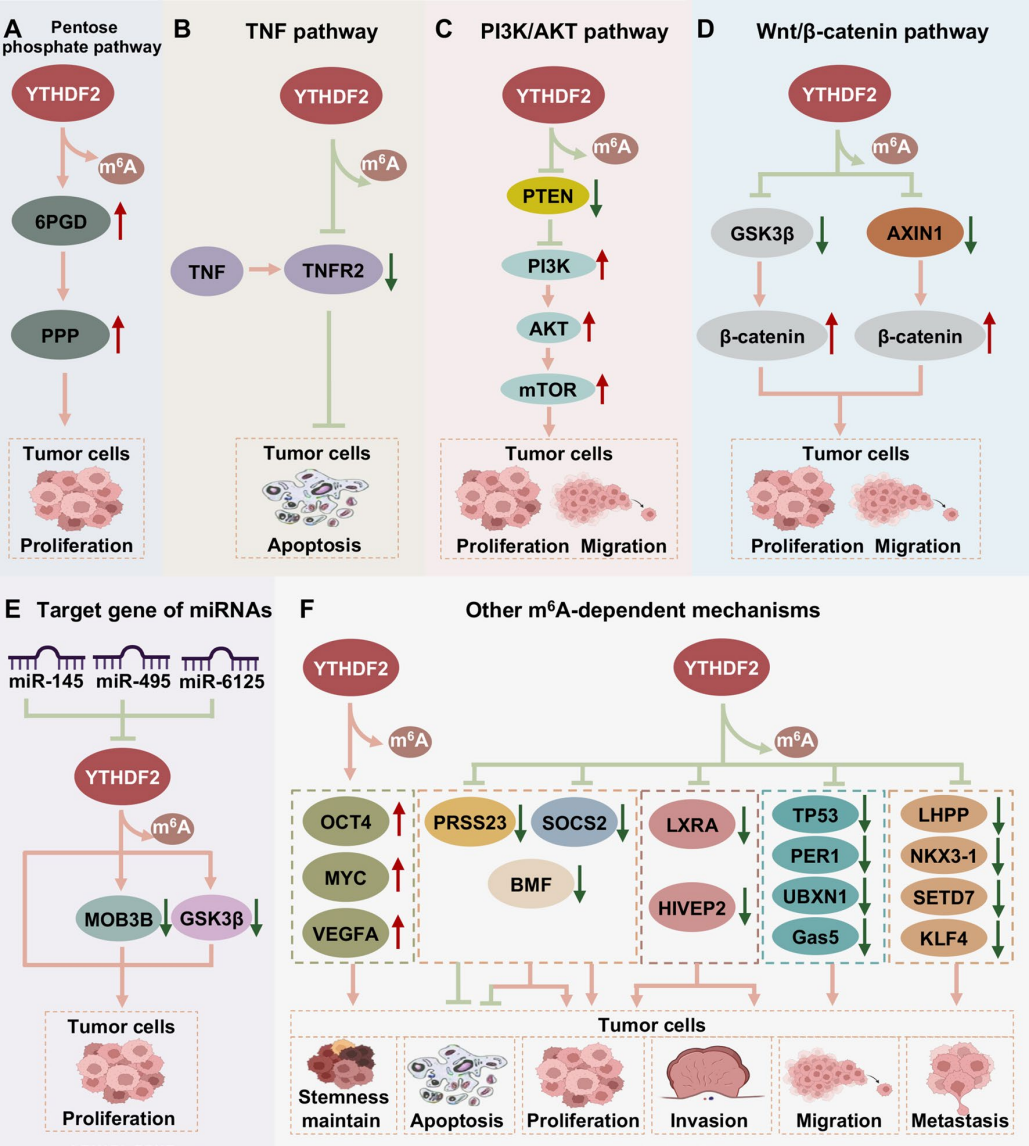
The underlying mechanisms of YTHDF2 in promoting cancer progression. YTHDF2 plays a significant role in tumor proliferation, invasion, migration, metabolism, and apoptosis in an m^6^A-dependent manner. The fundamental mechanisms are shown as follow: **A** the pentose phosphate pathway, **B** tumor necrosis factor (TNF) signaling, **C** the PI3K/AKT signaling pathway, **D** Wnt/β-catenin pathway, **E** miRNAs modulate YTHDF2 expression, **F** YTHDF2 modulates the expression of tumor suppressors in an m^6^A-dependent manner

**Fig. 4:**
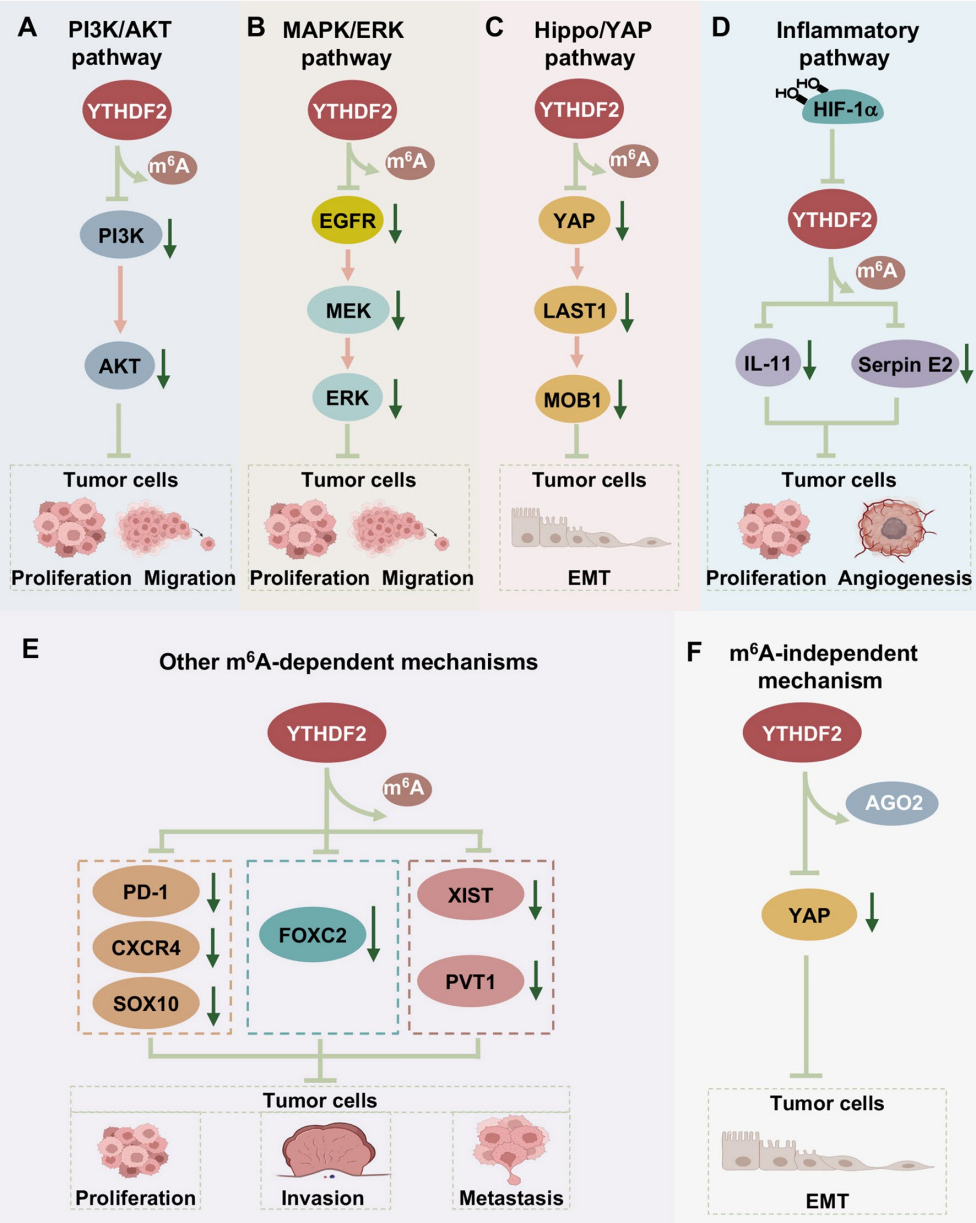
Roles of YTHDF2 in inhibition of cancer progression. YTHDF2 plays an essential role in tumor proliferation, invasion, migration, and metastasis. The fundamental mechanisms are shown as follow: **A** the PI3K/AKT signaling pathway, **B** the MAPK/ERK pathway, **C** Hippo/YAP pathway, **D** the inflammatory pathway, **E** YTHDF2 regulates the degradation of oncogenes in an m^6^A-dependent manner, **F** YTHDF2 directly interacts with YAP to enhance its degradation

As stated above, YTHDF2 is linked to multiple functions of human cancer cells and acts as an oncogene or tumor suppressor gene in different cancers. Here, the associated mechanisms of YTHDF2 in human cancers are listed and separated by its different expression patterns in various tumors. As presented in Fig. 3, the underlying mechanisms of YTHDF2 as an oncogene in various malignancies are gathered and summarized. YTHDF2 is also diminished and acts as a tumor suppressor in other malignant tumors. The underlying molecular mechanisms of YTHDF2 as a tumor suppressor were collected and are shown in Fig. 4.

### YTHDF2 regulated the PPP

The pentose phosphate pathway (PPP) plays a significant role in regulating the growth of tumor cells by providing cells with ribose-5-phosphate and NADPH with the help of its key regulatory enzyme glucose-6-phosphate dehydrogenase (G6PD) and its rate-limiting enzyme 6-phosphogluconate dehydrogenase (6PGD) [79]. Sheng et al. identified that YTHDF2 could directly bind to the m^6^A-modified site of the 3'-UTR of 6PGD and accelerate the translation of 6PGD mRNA in lung cancer cells. Consequently, the expression of 6PGD in epigenetics was increased to enhance the flux of PPP and promote the cellular metabolism and tumor growth of lung cancer (Fig. 3A).

### YTHDF2 suppressed TNF signaling pathway

As was reported, silencing of YTHDF2 could increase the half-life of the m^6^A levels of mRNA, indicating that YTHDF2 accelerated the degradation of m^6^A mRNA in leukemia [38]. Interestingly, the role of YTHDF2 was also found to be related to inhibition of tumor necrosis factor (TNF), which subsequently suppressed apoptosis of tumor cells. Specifically, knockdown of YTHDF2 was found to lead to the upregulation of TNF receptor superfamily member 1b (TNFRSF1B), which encodes TNF receptor 2 (TNF-R2), by extending the half-life of m^6^A-modified TNFRSF1B transcripts in leukemia cells [38, 63]. Previous studies indicated that TNFR2 binds to its ligand TNF, to mediate the binding of some adaptor proteins, which in turn initiate signal transduction to regulate cell death [80]. Thus, YTHDF2 may take part in inhibiting cell apoptosis by TNF signaling (Fig. 3B).

### YTHDF2 regulated the PI3K/AKT signaling pathway

Activated phosphoinositide-3-kinase (PI3K) triggers AKT activation, leading to the activation of mammalian target of rapamycin (mTOR) and other signaling pathways that promote cell survival [81, 82], which is inhibited by PTEN [83]. Analysis of the RIP assay indicated that YTHDF2 could recognize and bind to PTEN mRNA to promote its degradation [39]. Therefore, the decreased expression of PTEN promotes the activation of PI3K/AKT signaling, thereby contributing to tumorigenesis (Fig. 3C). PI3K and PTEN modulated the downstream AKT and other effectors by acting contrary roles as ‘on–off’ switches [84]. Phosphoinositide-3-kinase catalytic beta (PI3KCB), a catalytic subunit of PI3K, was reported to be modified by m^6^A resulting in its degradation, which was caused by YTHDF2, and subsequently inhibited the activation of PI3K/AKT signaling pathway to limit tumor progression (Fig. 4A) [57].

### YTHDF2 regulated the Wnt/β-catenin pathway

The Wnt/β-catenin pathway is highly conserved, and its abnormal activation facilitates cancer progression by enhancing cell proliferation and metastasis [85, 86]. Glycogen synthase kinase 3 beta (GSK3β) is a crucial component of the Wnt/β-catenin pathway, and its inactivation causes β-catenin to concentrate in the cell and transfer to the nucleus to enhance the progression of tumors [87, 88]. YTHDF2 was reported to recognize and bind m^6^A-modified GSK3β mRNA to promote its degradation, which subsequently decreased the phosphorylation of β-catenin to enhance the stability of the β-catenin protein, thereby promoting CRC cell proliferation [49]. Additionally, AXIN1, encoding a negative regulator of the Wnt/β-catenin pathway, was identified as a direct target of YTHDF2. Specifically, a remarkable enrichment of m^6^A in AXIN1 mRNA was detected, and the interaction between YTHDF2 and AXIN1 was determined by RIP-qPCR. Overexpression of YTHDF2 shortened the half-life of AXIN1 to decrease its expression to promote Wnt/β-catenin signaling, thus enhancing the progression of lung cancer (Fig. 3D) [62].

### YTHDF2 acted as the target gene of miRNAs

It has been reported that miRNA is a significant bioactive molecule that induces posttranscriptional gene regulation in eukaryotes [89]. Yang et al. elaborated that miR-145 could modulate the levels of m^6^A by targeting the 3'-untranslated region (3'- UTR) of YTHDF2 mRNA in HCC cells [55]. Moreover, YTHDF2, which was negatively linked to miR-145, could reduce the levels of m^6^A in HCC cells, thereby promoting the proliferation of HCC cells [55]. Similarly, YTHDF2 was found to be the direct target gene of miR-495 by the dual-luciferase reporter assay. Overexpressed YTHDF2 could reverse the inhibitory role of miR-495 and decreased the m^6^A levels of mps one binder kinase activator 3B (MOB3B) to promote the proliferation and migration of prostate cancer cells[67]. Moreover, miR-6125 could inhibit the proliferative ability of CRC cells by targeting the YTHDF2 mRNA [49]. In summary, YTHDF2 could act as a target of miRNA to participate in the progression of cancer. However, the relationship between YTHDF2 and other miRNAs in human malignancies requires further investigation (Fig. 3E).

### YTHDF2 inhibited the MAPK/ERK pathway

The mitogen-activated protein kinase (MAPK) / extracellular regulated kinase (ERK) signaling pathway is known to transmit signals from receptors on the cell surface to DNA in the nucleus, thereby promoting the proliferation and differentiation of cells [90, 91]. It was demonstrated that high expression of YTHDF2 in liver cancer cells could significantly reduce the phosphorylation levels of ERK and mitogen-activated protein kinase kinase (MAPKK/MEK) [36]. Furthermore, epidermal growth factor receptor (EGFR), one of the most important upstream targets of the MAPK/ERK pathway, is reported to be closely linked to cancer progression [36]. YTHDF2 was indicated to significantly suppress the expression of EGFR. Therefore, YTHDF2 could display a negative effect in regulating the stability of EGFR and then affect the MAPK/ERK pathway, which inhibits the growth of cancer (Fig. 4B) [36].

### YTHDF2 inhibited Hippo/YAP pathway

The Hippo signaling pathway acts a significant role in mediating cell division, proliferation, differentiation, and apoptosis. YES-associated protein (YAP) is an effector of this pathway, which could initiate the gene transcription and translation of the Hippo pathway [92, 93]. YTHDF2 was found to reduce the levels of the classical Hippo signal transduction factors, including YAP, LATS1 and Mob1 [56]. Besides, YAP was found to be frequently linked to EMT [94]. Knockdown of YTHDF2 could decrease the expression of E-cadherin to increase the expression of vimentin and Snail, which are associated with EMT [95]. Furthermore, YAP was positively correlated with vimentin levels and negatively correlated with E-cadherin in the TCGA database [96]. Taken together, YTHDF2 could modulate the Hippo/YAP signaling pathway to inhibit EMT, thereby suppressing tumor migration and invasion in some cancers (Fig. 4C).

### YTHDF2 regulated inflammatory cancer progression

Clinically, the stability of hypoxia-inducible factor (HIF) is linked to poor survival in various cancers [97, 98]. It has been reported that the activity of the YTHDF2 promoter could be reduced in hypoxia and reversed by the application of a HIF-2α inhibitor, which indicated the relationship between YTHDF2 and HIF-2α [50]. Moreover, silencing of YTHDF2 induced by HIF-2α could enhance the phosphorylation of signal transducer and activator of transcription 3 (STAT3) and the expression of interleukin-11 (IL-11) and serpin peptidase inhibitor clade E member 2 (Serpin E2) [50]. IL-11 could promote STAT3 activation and inflammatory cancer progression in an autocrine manner, and Serpin E2 could promote the progression of invasion and metastasis by reprogramming the tumor vascular system [99]. The results revealed that HIF-2α could induce hypoxia to reduce the expression of YTHDF2. In addition, YTHDF2 could inhibit the phosphorylation of STAT3 and the expression of Serpin E2, thereby reducing tumor growth and angiogenesis and resisting the occurrence of inflammatory cancer progression (Fig. 4D).

### Other m^6^A-dependent mechanisms of YTHDF2

YTHDF2 could enhance the degradation of oncogenes or tumor suppressor genes in an m^6^A-dependent manner, thereby influencing the development of tumors [23]. Chen et al. noted that YTHDF2 could bind to plasmacytoma variant translocation 1 (PVT1), a well-known oncogenic long noncoding RNA (lncRNA) in osteosarcoma. Furthermore, knockdown of YTHDF2 in cancer cells was shown to attenuate the upregulation of PVT1 and reversed the half-life of PVT1, indicating that YTHDF2 was vital to the stability of PVT1 and affected the progression of tumors [41]. It was also reported that high expression of YTHDF2 increased the decay of programmed death 1 (PD-1) mRNA, which played a significant role in melanoma. In addition, YTHDF2 could inhibit the progression of melanoma by suppressing the autophagy/NF-κB/FTO axis (Fig. 4E) [31]. In contrast, Chen et al. noted that in HCC, the 3'-end of SOCS2 transcript could directly bind to YTHDF2 [54]. Downregulation of YTHDF2 increased the expression of SOCS2 and negatively regulated the JAK/STAT signaling pathway, which suppressed the phosphorylation of STAT5 and inhibited the growth of cancer cells [54, 100]. Interestingly, luciferase assays and polysome profiling found that YTHDF2 retained the m^6^A methylation of the 5'-UTR of OCT4 mRNA, resulting in enhanced protein expression, thereby promoting liver cancer progression. Thus, YTHDF2 may function by regulating the expression of target genes to influence tumor development (Fig. 3F).

### m^6^A-independent manners of YTHDF2 in cancers

YTHDF2 could modulate the degradation of some mRNAs containing m^6^A [34]. As reported by Jin et al., YTHDF1 and YTHDF2 competitively interact with YTHDF3 to regulate the expression of YAP in lung cancer in a manner independent of m^6^A [59]. Moreover, YTHDF2 could also accelerate the degradation of YAP mRNA through the Argonaute 2 (AGO2) system, inhibiting the growth and metastasis of tumor cells to diminish disease progression (Fig. 4F) [59].

## Conclusions

In this review, we summarized the expression of YTHDF2 in human malignancies and generalized its relevant biological functions. More importantly, the underlying molecular mechanism and the clinical prognostic and therapeutic value of YTHDF2 in several cancers were also discussed. YTHDF2 was found to be highly expressed in multiple tumor tissues and cells, thereby acting as a carcinogenic factor [38, 54]. However, contrary conclusions were reported in melanoma, osteosarcoma, and CRC, where YTHDF2 acted as a tumor suppressor [31, 41, 47]. YTHDF2 was also found to be both upregulated and downregulated in lung cancer, GC, and liver cancer, which indicated that YTHDF2 might play a dual role as both an oncogene and tumor suppressor [39, 40, 45, 50, 54, 59]. The outcomes of the diversity may be linked to the extracellular microenvironment, heterogeneity of tumor tissues, and the related upstream or downstream regulators [101–104]. Additionally, the possible explanation may also be the interaction and the functioning site between YTHDF2 and the target genes. YTHDF2 could accelerate tumor growth by combining with tumor suppressors to trigger a downstream cascade, while it could exert the opposite effect by interacting with oncogenes [41, 65]. Therefore, further investigations are still needed to clarify the discrepancies to obtain better identifications of the effects and the underlying mechanisms of YTHDF2 in different cancers.

YTHDF2 can be involved in the progression of multiple cancers in an m^6^A-dependent manner, which is associated with various molecules and pathways [64]. MiR-145 and miR-495 could directly target YTHDF2 to affect the development of malignant tumors [55, 67]. Dysregulation of YTHDF2 in cancer could also regulate EMT, glucose metabolism, and apoptosis, which have been considered as significant factors in the progression of cancer [38, 40, 59]. Additionally, YTHDF2 enhances the degradation of oncogenes or tumor suppressors, such as the MAPK/ERK and PI3K/AKT signaling pathways, in an m^6^A-dependent manner. YTHDF2 was also found to take effects in an m^6^A-independent manner by promoting the degradation of YAP mRNA by the AGO2 system [59]. These reports suggest that YTHDF2 has tremendous potential in clinical application as a new target of diagnosis, treatment, and prognosis in tumor patients. Still, the reverse effect of YTHDF2 in distinct cancers or even in identical cancer might be related to genes with contrary functions or distinct binding sites, and the specific mechanism remains to be further elucidated.

In recent years, the role of m^6^A methylation in the prophylaxis and treatment of malignant tumors has received growing attention [105]. m^6^A methylation and its regulatory proteins were found to have the potential to be prognostic markers and therapeutic targets [106]. Several studies have shown that m^6^A methylation inhibitors, such as an inhibitor of FTO, can provide beneficial effects on the treatment of cancer [107]. As a primary “reader” protein of m^6^A, YTHDF2 has been shown to play a crucial role in m^6^A methylation modification [108, 109]. Given the above investigations, we summarized the significant effects of YTHDF2 on the modification of m^6^A and cancer progression. It can be conjectured that the development of effective inhibitors of YTHDF2 may provide novel strategies for the treatment of a variety of cancers in the future. However, the development and therapeutic effects of YTHDF2-related products still need to be further explored in the direction of cancer treatment.

## Data Availability

Not applicable.

## Abbreviations

6PGD: 6-Phosphogluconate dehydrogenase
AGO2: Argonaute 2
ALKBH5: AlkB homolog 5
AML: Acute myelocytic leukemia
BMF: Bcl2 modifying factor
BNIP3: BCL2 interacting protein 3
CC: Cervical cancer
ccRCC: Clear cell renal cell carcinoma
CRC: Colorectal cancer
CSC: Cancer stem cells
CSF-1: Colony stimulating factor 1
CXCR4: C-X-C motif chemokine receptor 4
EGFR: Epidermal growth factor receptor
eIF3: Eukaryotic initiation factor 3
EMT: Epithelial–mesenchymal transition
ERK: Extracellular regulated kinase
FOXC2: Forkhead box protein C2
FTO: Fat mass and obesity associated-protein
G6PD: Glucose-6-phosphate dehydrogenase
GAS5: Growth arrest specific 5
GBM: Glioblastoma
GC: Gastric cancer
GEO: Gene Expression Omnibus
GSEA: Gene Set Enrichment Analysis
GSK3β: Glycogen synthase kinase 3 beta
HCC: Hepatocellular carcinoma
HIF: Hypoxia-inducible factor
HIVEP2: HIVEP zinc finger 2
HNRNPs: Heterogeneous nuclear ribonucleoprotein protein families
HNSCC: Head and neck squamous cell carcinoma
IGF2BPs: Insulin-like growth factor 2 mRNA binding protein families
IL-11: Interleukin-11
KIAA1429: VIRMA, Vir like m^6^A methyltransferase associated
KLF4: Kruppel like factor 4
LHPP: Phospholysine phosphohistidine inorganic pyrophosphate phosphatase
lncRNAs: Long noncoding RNAs
LXRA: Liver X receptors A
m^6^A: N6-methyladenosine
MAPK: Mitogen-activated protein kinase
MAPKK/MEK: Mitogen-activated protein kinase kinase
METTL: Methyltransferase like
miRNAs: MicroRNAs
MOB3B: MOB kinase activator 3B
mRNAs: Messenger RNAs
MTC: Methyltransferase complex
mTOR: Mammalian target of rapamycin
NKX3–1: NK3 homeobox 1
NSCLC: Non-small-cell lung cancer
OCT4 (POU5F1): POU class 5 homeobox 1
OS: Overall survival
PC: Pancreatic cancer
PCa: Prostate cancer
PD-1: Programmed death 1
PER1: Period circadian regulator 1
PI3K: Phosphoinositide-3-kinase
PIK3CB: Phosphoinositide-3-kinase catalytic beta
PPP: Pentose phosphate pathway
PRSS23: Serine protease 23
PTCL-NOS: Peripheral T-cell lymphoma, not otherwise specified
PTEN: Phosphate and tension homology deleted on chromosome ten
PVT1: Plasmacytoma variant translocation 1
RBM15/15b: RNA binding motifprotein 15/15b
RFS: Relapse-free survival
Serpin E2: Serpin peptidase inhibitor clade E member 2
SETD7: SET domain containing 7
SOCS2: Suppressor of cytokine signaling 2
SOX10: SRY-Box transcription factor 10
STAT: Signal transducers and activators of transcription
STAT3: Signal transducer and activator of transcription 3
TCGA: The Cancer Genome Atlas
TNBC: Triple-negative breast cancer
TNF: Tumor necrosis factor
TNF-R2: TNF receptor 2
TNFRSF1B: TNF receptor superfamily member 1b
TP53: Tumor protein P53
UBXN1: UBX domain protein 1
VEGFA: Vascular endothelial growth factor A
WTAP: Wilms tumor 1-associated protein
XIST: X inactivate-specific transcript
YAP: YES-associated protein
YTHDF: YT521-B homology domain family
ZC3H13: Zinc finger CCCH-type containing 13
